# The Relationship between the Efficacy of Tonsillectomy and Renal Pathology in the Patients with IgA Nephropathy

**DOI:** 10.1155/2014/451612

**Published:** 2014-05-19

**Authors:** Tsutomu Nomura, Yoshimi Makizumi, Tsuyoshi Yoshida, Tatsuya Yamasoba

**Affiliations:** Department of Otolaryngology and Head and Neck Surgery, Faculty of Medicine, University of Tokyo, 7-3-1 Hongo, Bunkyo-ku,Tokyo 113-8655, Japan

## Abstract

*Objective.* The aim of this study was to evaluate the effects of tonsillectomy as a treatment for IgA nephropathy in relation to renal pathological findings. *Methods.* This is a retrospective analysis of 13 patients having IgA nephropathy treated by tonsillectomy. *Results.* UP/UCre levels decreased from 820.8 to 585.4 one month postsurgery and then showed slight worsening to 637.3 at the most recent follow-up. There was no significant difference in the improvement rate between pathological grades I–III and IV. There was positive correlation between Pre-UP/UCre level and the reduction rate of UP/UCre, which was statistically significant (*R* = 0.667, *R* ^2^ = 0.445, and *P* = 0.01). *Conclusions.* Reduction of UP/UCre at one month postsurgery is considered to be an overall prognostic factor, and tonsillectomy is considered to be an effective therapy for IgA patients regardless of the grade of renal pathology.

## 1. Introduction

IgA nephropathy (IgAN) is the most common form of chronic glomerulonephritis with IgA deposits present mainly in the mesangial areas in Japan. Several retrospective studies have investigated the effect of tonsillectomy on IgA nephropathy [1–3]. A great deal of data regarding the effect of tonsillectomy on patients with IgA nephropathy has been reported, although few reports have examined the relationship between the efficacy of tonsillectomy and renal pathology.

In terms of renal pathology, Akagi and Nishizaki [4] recommended tonsillectomy for IgA patients with grade I to III renal pathology. Xie et al. [5] reported that tonsillectomy was not effective in IgA patients with marked renal damage.

The purpose of this study is to evaluate the effects of tonsillectomy as a treatment for IgAN in relation to renal pathological findings.

## 2. Materials and Methods

We performed a retrospective review of 13 patients, 4 males and 9 females, with IgAN referred from the nephrology department of our university hospital. All patients underwent tonsillectomy (Table 1). The age at tonsillectomy was 30.3 years on average (range: 13 to 65 years). Mean follow-up interval was 186 months from the first visit and 19 months from tonsillectomy. Three patients had steroid pulse therapy after tonsillectomy. Almost all patients had medication of angiotensin converting enzyme inhibitor (ACEI), angiotensin II receptor blocker (ARB), and diuretics.

Renal biopsy findings were classified in terms of prognosis as good (grade I), relatively good (grade II), relatively poor (grade III), and poor (grade IV) using the criteria of the Committee of IgA Nephropathy—the Special Study Group of Progressive Glomerular Disease, the Ministry of Health, Labor and Welfare of Japan [6]. Through renal biopsy, 4, 3, 1, and 5 patients were classified as grades I, II, III, and IV, respectively.

Criteria of IgA pathology are as follows.

 Grade I: slight mesangial proliferation and increased matrix were observed. No glomerulosclerosis, crescent formation, or adhesion to Bowman's capsules was observed. No prominent changes were seen in the interstitium, renal tubuli, or blood vessels.
 Grade II: slight mesangial cell proliferation and increased matrix were observed. Glomerulosclerosis, crescent formation, or adhesion to Bowman's capsules was observed in less than 10% of all biopsied glomeruli. Interstitial and vascular findings were the same as in Group I.
 Grade III: moderate, diffuse mesangial proliferation and increased matrix were observed. Glomerulosclerosis, crescent formation, or adhesion to Bowman's capsules was observed in less than 10–30% of all biopsied glomeruli.
 Cellar infiltration was slight in the interstitium except around some sclerosed glomeruli. Tubular atrophy was slight, and mild vascular sclerosis was observed.
 Grade IV: severe, diffuse mesangial proliferation and increased matrix were observed. Glomerulosclerosis, crescent formation, or adhesion to Bowman's capsules was observed in more than 30% of all biopsied glomeruli. When sites of sclerosis are totaled and converted to global sclerosis, the sclerosis rate was more than 50% of all glomeruli. Some glomeruli also showed compensatory hypertrophy.

Presurgery, one month postsurgery, and most recent serous IgA level, serous creatinine, and extent of hematuria were reviewed. The rate of urine protein/urine creatinine (UP/UCre) and estimated glomerular filtration rate (eGFR) were calculated. The reduction rate of UP/UCre was calculated with the following formula.

### 2.1. (Pre-UP/UCre-Post-UP/UCre)/Pre-UP/UCre

For statistical analysis, Mann-Whitney* U* test was performed to compare the two means, *χ* ^2^ test was used for rate analysis, and regression analysis to determine correlation was carried out with SPSS ver.10.0.

## 3. Results

**Figure 1:**
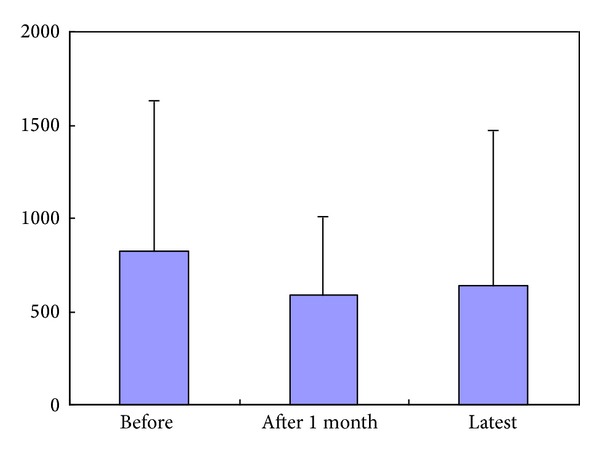
UP/UCre levels.

The data obtained before surgery, one month after surgery, and at most recent visit are presented in Table 2. Creatinine, IgA, eGFR, and hematuria levels exhibited virtually no change when compared to presurgery. UP/UCre levels decreased from 820.8 (presurgery) to 585.4 one month postsurgery and then showed slight increase to 637.3 at the most recent visit, but the difference was not statistically significant (Figure 1). The change was not related to the presence of postoperative steroid pulse therapy.

The improvement rate one month postsurgery compared to presurgery in terms of renal pathology is shown in Table 3. The UP/UCre level was reduced in 2, 2, 1, and 2 patients classified as grades I, II, III, and IV, respectively. Hematuria was improved in 2, 1, 1, and 1 patients in grades I, II, III, and IV, respectively. Serous IgA was improved in 3, 2, and 2 patients in grades I, II, and IV, respectively.

The improvement rate at the most recent visit compared to presurgery is presented also in Table 3. Hematuria showed slight improvement in all grades, but levels of other factors remained almost the same. There was no significant difference in the improvement rate between pathological grades I–III and IV.

**Figure 2:**
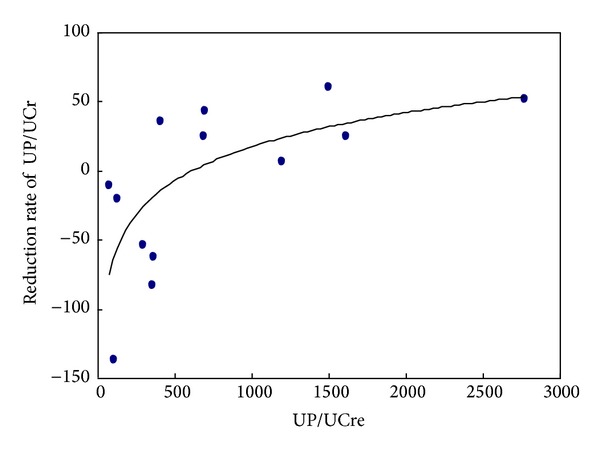
The correlation between UP/UCre and the reduction rate of UP/UCre.

The correlation between UP/UCre and the reduction rate of UP/UCre is plotted in Figure 2. The Pre-UP/UCr had a positive correlation with the reduction rate of UP/UCre, with a statistical significance (*R* = 0.667, *R* ^2^ = 0.445, and *P* = 0.01).

## 4. Discussion

IgA nephropathy (IgAN) is the most common form of chronic glomerulonephritis with IgA deposits present mainly in the mesangial areas in Japan [6]. Tonsillitis is believed to play an important role in the pathogenesis in IgAN. Several retrospective studies have investigated the effect of tonsillectomy on IgA nephropathy [1–3]. Although tonsillectomy has been recommended for patients exhibiting grade I to III renal pathology, not those exhibiting grade IV [5], the current study suggested that an improvement in the extent of urine protein, IgA, and hematuria can be achieved also in patients with grade IV renal pathology.

The UP/UCre level is presumed to reflect daily protein excretion dose and therefore state of renal function. The UP/UCre level was decreased one month postsurgery, and although it showed slight worsening at the most recent visit, it remained to be improved when compared to presurgery.

In terms of the correlation between the UP/UCre level and the reduction rate of UP/UCre, the Pre-UP/UCre level had positive correlation with the reduction rate of UP/UCre. This suggests that the effect of tonsillectomy is not related to the clinical stage of IgAN. Thus, the reduction of UP/UCre level at one month postsurgery is considered to be an overall prognostic factor, and tonsillectomy is considered to be an effective therapy for IgA patients regardless of the grades of renal pathology.

## 5. Conclusion

Reduction of UP/UCre level one month after tonsillectomy is considered to be an overall prognostic factor, and tonsillectomy is considered to be an effective therapy for IgA regardless of renal pathological grades.

## Figures and Tables

**Table 1:** Baseline characteristic of 13 patients with IgA nephropathy.

Gender (male/female)	4/9
Age at tonsillectomy	30.3 (13–65) years
Follow-up interval	
After the first visit	186 m
After tonsillectomy	19 m

**Table 2:** Overall examined data.

	Presurgery	One month PS*	Recent
Cre	0.8 ± 0.3	0.8 ± 0.3	0.8 ± 0.3
IgA	242.2 ± 67.5	212.7 ± 64.0	222.3 ± 77
eGFR	90.1 ± 29.6	89.3 ± 30.7	86.4 ± 30.5
UP/UCre	820.8 ± 813.5	585.4 ± 427.2	637.3 ± 832.1
Hematuria	10/13	10/13	10/13

*PS: postsurgery.

**Table 3:** The improvement rate one month postsurgery compared to presurgery in terms of renal pathology.

	UP/UCre	Hematuria	IgA
Grade			
I	2/4	2/4 (3/4)	3/3
II	2/3	1/3	2/3
III	1/1	0/1	1/1
IV	2/5	1/5 (2/5)	2/4

*( ): data at most recent.
